# Necroptosis of nucleus pulposus cells involved in intervertebral disc degeneration through MyD88 signaling

**DOI:** 10.3389/fendo.2022.994307

**Published:** 2022-09-21

**Authors:** Hong Fan, Zhe Chen, Hai-Bin Tang, Le-Qun Shan, Zi-Yi Chen, Shi-Chang Liu, Yong-Yuan Zhang, Xin-Yu Guo, Hao Yang, Ding-Jun Hao

**Affiliations:** ^1^ Shaanxi Spine Medicine Research Center, Translational Medicine Center, Department of Spine Surgery, Hong Hui Hospital, Xi’an Jiaotong University, Xi’an, China; ^2^ Department of Neurology, The Second Affiliated Hospital of Xi’an Jiaotong University, Xi’an, China; ^3^ Department of Laboratory Medicine, Xi’an Central Hospital, Xi’an Jiaotong University, Xi’an, China; ^4^ Department of Endocrinology, The First Affiliated Hospital of Xi’an Jiaotong University, Xi’an, China

**Keywords:** ivdd, necroptosis, nucleus pulposus cells, MyD88 signaling, low back pain

## Abstract

**Background context:**

Low back pain, affecting nearly 40% of adults, mainly results from intervertebral disc degeneration (IVDD), while the pathogenesis of IVDD is still not fully elucidated. Recently, some researches have revealed that necroptosis, a programmed necrosis, participated in the progression of IVDD, nevertheless, the underlying mechanism remains unclear.

**Purpose:**

To study the mechanism of necroptosis of Nucleus Pulposus (NP) cells in IVDD, focusing on the role of MyD88 signaling.

**Study design:**

The expression and co-localization of necroptotic indicators and MyD88 were examined *in vivo*, and MyD88 inhibitor was applied to determine the role of MyD88 signaling in necroptosis of NP cells *in vitro*.

**Methods:**

Human disc specimens were collected from patients receiving diskectomy for lumbar disc herniation (LDH) or traumatic lumbar fractures after MRI scanning. According to the Pfirrmann grades, they were divided into normal (Grades 1, 2) and degenerated groups (4, 5). Tissue slides were prepared for immunofluorescence to assess the co-localization of necroptotic indicators (RIP3, MLKL, p-MLKL) and MyD88 histologically. The combination of TNFα, LPS and Z-VAD-FMK was applied to induce necroptosis of NP cells. Level of ATP, reactive oxygen species (ROS), live-cell staining and electron microscope study were employed to study the role of MyD88 signaling in necroptosis of NP cells.

**Results:**

*In vivo*, the increased expression and co-localization of necroptotic indicators (RIP3, MLKL, p-MLKL) and MyD88 were found in NP cells of degenerated disc, while very l low fluorescence intensity in tissue of traumatic lumbar fractures. *In vitro*, the MyD88 inhibitor effectively rescued the necroptosis of NP cells, accompanied by increased viability, ATP level, and decreased ROS level. The effect of MyD88 inhibition on necroptosis of NP cells was further confirmed by ultrastructure of mitochondria shown by Transmission Electron Microscope (TEM).

**Conclusion:**

Our results indicated that the involvement of MyD88 signaling in the necroptosis of NP cells in IVDD, which will replenish the pathogenesis of IVDD and provide a novel potential therapeutic target for IVDD.

## Introduction

Low back pain, affecting nearly 40% of adults in the world, mainly results from intervertebral disc degeneration (IVDD) (1, 2). Although discectomy could relief the pain for patients with surgical indications, there are no strategy could suppress the progression of the degeneration, partially due to un-fully elucidated pathogenesis of IVDD (3–6). Therefore, further clarification of the underlying mechanisms of IVDD is needed for developing novel and specific treatment to inhibit degeneration.

It is widely accepted that the death of nucleus pulposus (NP) cells plays important role in the progress of intervertebral disc degeneration (IVDD) (7, 8). Apoptosis has been considered as the main way of death of NP cells in IVDD, but the results were not very satisfactory from the treatment strategies based on apoptosis inhibition (9, 10), indicating that other types of cell death contribute to the IVDD.

Necroptosis, a form of programmed necrotic cell death, which is regulated by receptor-interacting protein kinase 3 (RIPK3) and mixed lineage kinase domain-like (MLKL), has been demonstrated to participate in the loss of NP cells in IVDD (11–14). It has been reported that compression could induce necroptosis of NP cells through mitochondrial dysfunction and endoplasmic reticulum stress *in vitro (* 15–17). In addition, Ding and his colleagues found that RIP3and MLKL markedly increased in severely degenerated disc tissues (14). Nevertheless, less attention has been paid to the mechanism of necroptosis of NP cells in degenerated disc. In our previous study, we have demonstrated that astrocytes underwent necroptosis partially *via* TLR/MyD88 signaling after spinal cord injury (18). MyD88 pathway was indeed participated in the progression of IVDD (19–22), however, whether that it involved in RIP3/MLKL mediated necroptosis of NP cells has never been studied.

To identify the role of MyD88 signaling in necroptosis of NP cells in IVDD, we extracted the degenerated and normal disc from patients by discectomy, and confirmed the necroptosis of NP cells and annulus fibrosus (AF) cells on the degenerated disc. We further demonstrated that inhibition of MyD88 could rescue the necroptosis of NP cells shown by increased viability, ATP level, ultrastructure of mitochondria, as well as the decreased ROS level.

In conclusion, our present study provides novel insights into the mechanism of necroptosis of NP cells in IVDD, which might be a potential target for the further therapy.

## Method and materials

### Clinical specimens

This study has been approved by the Ethics Committee of HongHui Hospital, Xi’an Jiaotong University. All participants in our study have signed written informed consent before surgery. All patients were evaluated by the magnetic resonance imaging (MRI) scans before surgery according to the Pfirrmann grades, and they were divided into normal (Grades I, II) and degenerated groups (IV, V) (23, 24). A total of 29 human lumber disc tissues (9 normal lumber disc samples were from patients of traumatic lumbar fractures, and the other 20 degenerated samples were from LDH patients) were harvested after discectomy. In the morphological analysis, 6 normal discs were included and 6 degenerated discs were randomly selected from the 17 degenerated samples. For western-blot analysis, another 3 normal discs and 3 degenerated discs were collected.

### Immunofluorescence

The discs were fixed by 4% paraformaldehyde (PFA) for 24h at 4°C, followed by dehydration in 25% sucrose solution for 48 h. The tissues were then implanted into optimal cutting temperature compound (Sakura), and serial 12-μm-thick sections were cut on freezing microtome (Leica). All sections were placed on slides and stored at -20°C for immunostaining.

After blocking with 0.01M PBS solution containing 3% BSA and 0.3% Triton X-100 for 30 min, the sections were incubated with primary antibodies in a humid chamber at 4°C overnight. Anti-RIP3(1:300, Santa Cruz Biotechnology, sc-374639), anti-MLKL(1:200, Milipore, MABC 604), anti- p-MLKL(1:200, Abcam, ab187091), and anti-MyD88(1:400, R&D, MAB2928) antibodies were used in this study. The slides were then washed by 0.01M PBS three times, and incubated with corresponding secondary antibodies. The nuclei were stained with Hoechst 33342. The images were photographed by confocal microscope (LSM 800, Zeiss).

### Western-blot analysis

Normal and degenerated discs were obtained to detect the expression of MyD88. Ice-cold radioimmunoprecipitation assay (RIPA, Boster) buffer containing 1% phenylmethylsulfonyl fluoride (PMSF, Boster) were applied to extract total protein. Sodium dodecyl sulfate-polyacrylamide gel (SDS-PAGE,10%) was utilized to separate proteins, which was then transferred to polyvinylidene fluoride (PVDF, Milipore) membrane. The membranes were then incubated with primary antibodies anti-MyD88 (R&D, MAB2928, 1:2000), β-actin (Cell Signaling Technology, 4970, 1:3500) at 4°C for 12h, followed by incubation with correspondence secondary antibodies at room temperature for 1.5h. The bands were visualized and analyzed by Bio-Rad Image Lab System.

### Microarray data collection and processing

Microarray data set (GSE70362) was obtained from NCBI Gene Expression Omnibus (GEO), which was used to analyze the correlation between MyD88 and different grade of IVDD. The GSE70362 data set was generated utilizing the GPL17810. The normalization and quality control of GSE70362 was processed with the ‘limma’ R package (version 3.42.2). The correlation between the expression of MyD88 and different grades of IVDD were performed by spearman method.

### Animal samples

All animals utilized in experiments were purchased form the Animal Center of Xi’an Jiaotong University. Animal procedures were approved by Animal Care and Use Committee of Xi’an Jiaotong University.

### Isolation, culture and treatment of rat NP cells

Sprague-Dawley rats (about 200g) were applied to obtain NP cells in this study. After removing the lumber spine, lumber discs were cut to get the NP tissues. Then tissues were chopped and digested for 15min in 0.25% type II collagenase (Sigma) at 37°C and gently shaken every 5 minutes during the digestion. After centrifuged at 1000 rpm for 5min, samples were suspended and cultured in DF12 (Gibco) containing 20% FBS (Gibco). Medium was changed every three days. When cells reached a confluence of 80-90%, they were digested and reseeded for further experiments.

According to our previous studies (18, 25), necroptosis of NP cells was induced by TLZ treatment (100 ng/ml of TNFα + 4 μg/ml of LPS + 20 μM of z-VAD) for 48 h. To explore the role of MyD88 in necroptosis, NP cells were treated with 100 μM of MyD88 inhibitory peptide (Novas, NBP2-29328) 8 h before TLZ challenge.

### Cell viability assay

NP cells were seeded into a 96-well plate (3×10^3^ cells/well) and treated by TLZ or TLZ + MyD88 inhibitor. The cell viability of NP cells were tested by Cell Counting Kit-8 (CCK-8, Sangon Biotech) according to the manual.

### ROS level measurement

For testing reactive oxygen species (ROS) levels, 10 μM of DCFH-DA probe (Sigma) was added into NP cells and incubated at 37°C for 30min. The fluorescence intensities were measured by a microplate reader (TECAN, Infinite M200).

### ATP examination

Cell Titer-Glo assay Kit (Promega) was used to examine ATP level according to the manual and our previous studies (26).

### Propidium iodide staining

After NP cells were treated with TLZ, TLZ + MyD88 inhibitor or MyD88 inhibitor, propidium iodide (PI) (Sigma) and Hoechst 33342 were supplied into the cells and incubated at 37°C for 20min. Then, cells were washed three times by PBS and fixed by 4% PFA for 15min at RT. Cells were photographed by confocal microscope (LSM 800, Zeiss).

### Ultrastructure of NP cells detection

The ultrastructure of NP cells was detected by transmission electron microscope. Briefly, NP cells were harvested and fixed in 4% PFA containing 0.05% glutaraldehyde for 20 min, and 50-μm sections were prepared with vibratome (VT 1000S, Leica). After immersed in 0.5% osmium tetroxide, the sections were dehydrated with graded ethanol series, and incubated with propylene oxide and embedded in Epon812. After contrasted in uranyl acetate, the sections were detected by electron microscope (JEM-1230).

### Statistical analysis

Quantification was performed by a person who is blind to this study. GraphPad prism 8.0 was used for statistical analysis. For multiple group comparisons, significance was assessed by one-way analysis of variance followed by *post hoc* Tukey’s analysis. All data were showed as mean ± standard deviation (SEM). P values of less than 0.05 were regarded as statistically significant.

## Results

### Detection of necroptosis in human disc tissues

**Figure 1:**
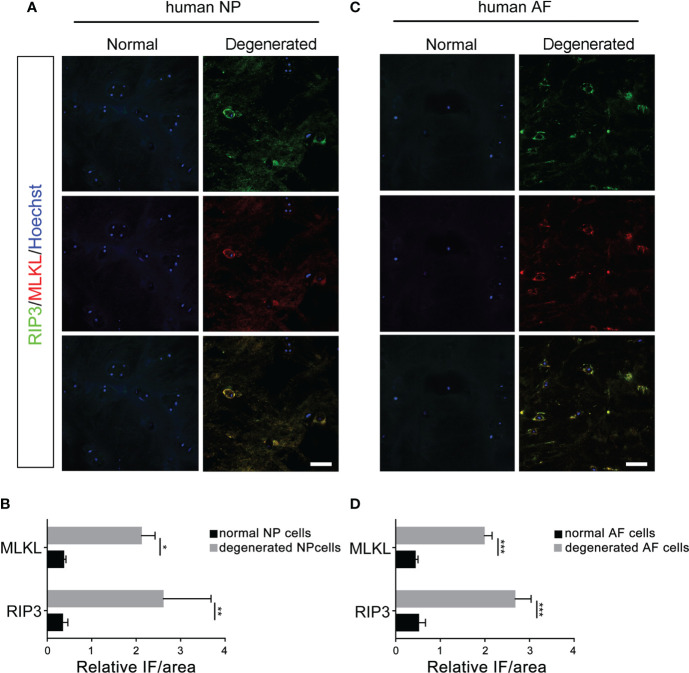
Analysis of necroptosis of NP cells and AF cells in the normal and degenerated disc. **(A)** Double-staining of RIP3 with MLKL was performed to detect necroptotic NP cells in normal and degenerated disc. **(B)** Relative IF intensity of MLKL and RIP3 in NP cells. **(C)** Double-staining of RIP3 with MLKL was performed to detect necroptotic AF cells in disc. **(D)** Relative IF intensity of MLKL and RIP3 in AF cells. *P < 0.05, **P < 0.01, ***P < 0.001. N = 6. Bars = 50μm.

**Figure 2:**
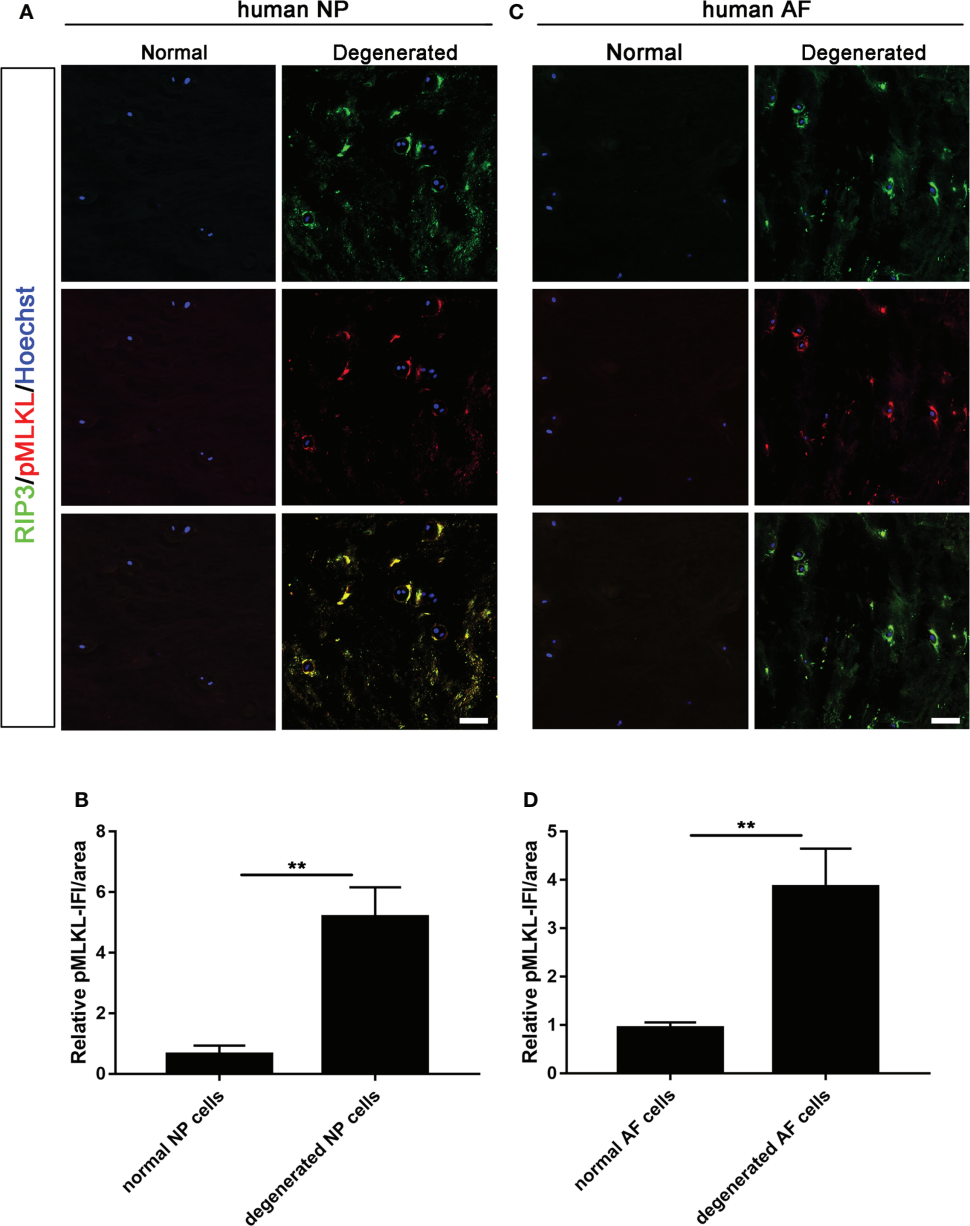
Analysis of necroptosis of NP cells and AF cells in the normal and degenerated disc. **(A)** Double-staining of RIP3 with p-MLKL was performed to detect necroptotic NP cells in normal and degenerated disc. **(B)** Relative IF intensity of p-MLKL in NP cells. **(C)** Double-staining of RIP3 with p-MLKL was performed to detect necroptotic AF cells in disc. **(D)** Relative IF intensity of p-MLKL in AF cells. **P < 0.01. N = 6. Bars = 50μm.

Double-immunostaining of RIP3/MLKL and RIP3/p-MLKL was performed to examine the necroptosis in degenerated and normal disc from patients undergoing discectomy. Our data showed that the expression levels of RIP3 and MLKL were significantly increased in NP cells of the degenerated disc, compared to normal (**Figures 1A, B**). The increased expression of RIP3 and p-MLKL was also found in NP cells of the degenerated disc (**Figures 2A, B**). Besides the NP cells, higher levels of RIP3, MLKL and p-MLKL were also detected in the annulus fibrosus (AF) cells in degenerated disc (**Figures 1C, D**, **2C, D**). The above results indicated that necroptosis could be found in both of AF cells and NP cells in degenerated disc. Considering that the important role of NP cells in progress of IVDD, we focused on the necroptosis of NP cells in the following study.

### The role of MyD88 signal in necroptosis of NP cells

**Figure 3:**
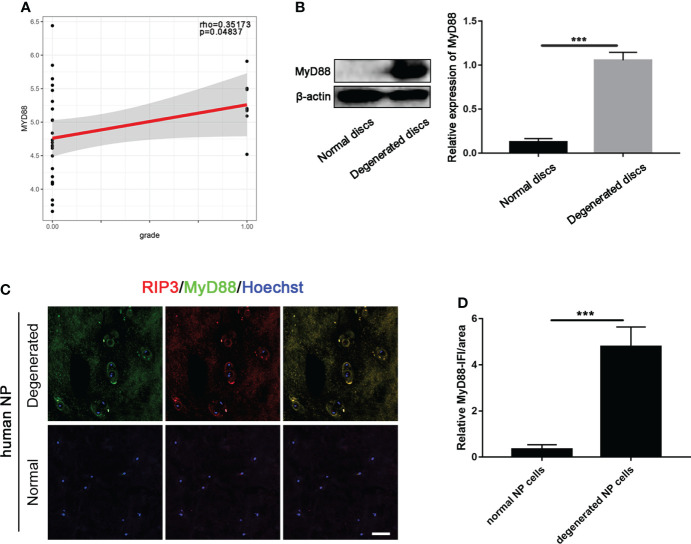
Expression of MyD88 in normal and degenerated disc. **(A)** The correlations of the expression of the MyD88 with IVDD grades by linear regression analysis. (“0.00” means early stage-thompson grade III, IV, “1.00” means advanced stage-thompson grade V). **(B)** Detection of MyD88 levels in normal and degenerated disc by western blot. ***P < 0.001. **(C)** Double-staining of RIP3 with MyD88 in NP cells in normal and degenerated disc. **(D)** Relative IF intensity of MyD88 in NP cells. ***P < 0.001. N = 6. Bar = 50μm.

We first conducted spearman test to determine the correlation between MyD88 and IVDD grades. Our data showed that MyD88 gene was positively correlated with the progression of IVD (MyD88: p = 0.04837, **Figure 3A**). Our data of western blot further showed that the expression of MyD88 was significantly increased degenerated discs, compared to normal discs (**Figure 3B**).

To further study the role of MyD88 in RIP3/MLKL-mediated necroptosis of NP cells, double-immunostaining of RIP3/MyD88 was performed. The co-localization of RIP3 and MyD88and higher expression level of MyD88 were found in NP cells of the degenerated disc (**Figures 3C, D**), indicating that MyD88 signal might participate in necroptosis of NP cells.

**Figure 4:**
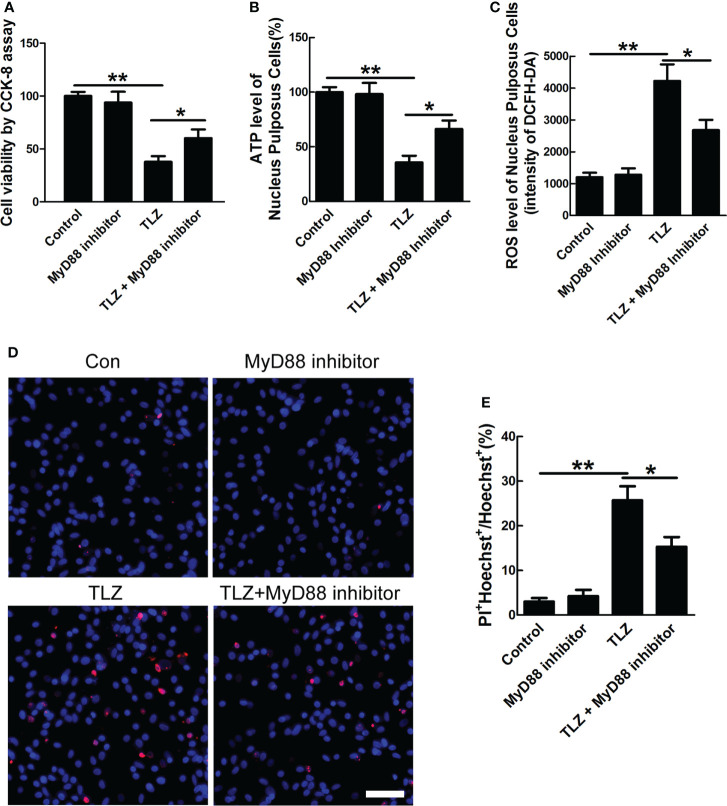
Effects of MyD88 signaling on necroptotic NP cells. **(A)** NP cells viability under TLZ with MyD88 inhibitor condition. **(B)** ATP level of necroptotic NP cells with blocking MyD88 signaling. **(C)** ROS level of NP cells with different conditions. **(D)** PI-positive cells under TLZ condition with or without MyD88 inhibitor. **(E)** Relative percentage of PI+ NP cells. *P < 0.05, **P < 0.01. N= 3 independent replicates. Bar = 50μm.

To further define the role of MyD88 signal in necroptosis of NP cells, MyD88 inhibitor was applied. The *in vitro* necroptosis of NP cells was established by TLZ treatment according to our previous study (18, 25). The data of CCK-8 assays showed that inhibiting MyD88 could significantly rescue decreased cell viability, ATP level and increased ROS level in NP cells upon TLZ treatment, while MyD88 inhibitor alone did not influence the viability, ATP level and ROS level of NP cells (**Figures 4A–C**). Besides these indicators of necroptosis, data of PI staining directly showed that MyD88 inhibitor could decrease TLZ-induced necrosis of NP cells (**Figures 4D, E**). The above data suggested that MyD88 signaling played a pivotal role in necroptosis of NP cells.

### Detection of the change of mitochondrial ultrastructure

**Figure 5:**
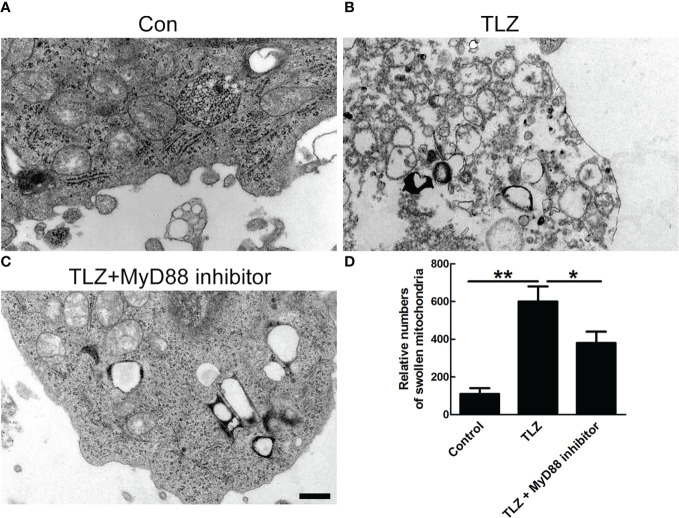
TEM photographs of ultrastructure of NP cells showing mitochondria. **(A)** Normal NP cells. **(B, C)** Normal NP cells under TLZ condition with or without MyD88 inhibitor. **(D)** Relative numbers of swollen mitochondria. *P < 0.05, **P < 0.01. N= 3 independent replicates. Bar = 1μm.

Considering that necroptosis was related to mitochondrial dysfunction, mitochondrial ultrastructure was detected by transmission electron microscope (TEM). The data showed that MyD88 inhibitor could significantly decrease the TLZ-induced mitochondria destruction, indicating that MyD88 inhibitor suppressed the necroptosis of NP cells partially *via* protecting mitochondria (**Figures 5A–D**).

## Discussion

Necroptosis has been demonstrated to play pivotal roles in diverse conditions, including traumatic and degenerative diseases (12, 18, 25). Recently, although necroptosis of NP cells has been reported *in vitro (* 15, 17), there still lacks of evidence that necroptosis of NP cells could occur in degenerated disc. In the present study, we confirmed the necroptosis of NP cells in degenerated human disc and demonstrated that necroptosis of NP cells involved in IVDD through MyD88 signaling. Our study referring to NP cells necroptosis may provide a promising target for IVDD therapy.

Although ferroptosis and apoptosis of AF cells had been investigated in IVDD (27–29), there is no reported study on necroptosis of AF cells in IVDD. In this study, increased expression levels of RIP3, MLKL and p-MLKL were also been found in AF cells, which indicated that AF cells also underwent necroptosis in degenerated disc. Considering that the loss of AF cells contributed to the degeneration of AF tissue, we speculate that necroptosis of AF cells might also participate in IVDD. The mechanism of AF cells necroptosis and its interaction with the death of NP cells in IVDD is worth to be explored in future.

Multiple Toll-like receptors(TLRs) are activated upon inflammatory stimulus. Each TLR selectively recruits specific adaptor proteins, and MyD88 is a common adapter shared by all known TLRs (30). Considering that Toll-like receptors activate necroptosis through RIP3 pathway (31), we then focused on the role of MyD88 in necroptosis of NP cells. MyD88 signaling has been demonstrated to contribute to necroptosis in spinal cord injury, colitis and acute pancreatitis (18, 32, 33). In the present study, we demonstrated that MyD88 signaling also participate in necroptosis of NP cells, shown by the changed levels of ROS, ATP and mitochondrial structure. One of the limitations of this study is that rat NP cells was applied to investigate the role of MyD88 signaling in necroptosis, human NP cells will be utilized to clarify the role of MyD88 signaling in necroptosis of NP cells.

Although the role of MyD88 signaling in necroptosis of NP cells has been explored in the present study, the mechanism of necroptosis of NP cells in IVDD was still not fully clarified. Previously, we demonstrated that necroptotic astrocytes could release high mobility group box 1(HMGB1), which driven downstream inflammation in spinal cord injury (34). It was reported that HMGB1 was crucial in the progression of IVDD (19, 35–38). Therefore, we speculated that HMGB1 might be released from necroptotic NP cells and might be a participant in the progression of IVDD. The further clarification of the how necroptotic NP cells participate in IVDD needs to be explored in future.

## Conclusion

Necroptosis of Nucleus Pulposus Cells involved in intervertebral disc degeneration through MyD88 signaling.

## Data availability statement

The raw data supporting the conclusions of this article will be made available by the authors, without undue reservation.

## Ethics statement

The studies involving human participants were reviewed and approved by Ethics Committee of HongHui Hospital, Xi’an Jiaotong University. Written informed consent to participate in this study was provided by the participants’ legal guardian/next of kin. The animal study was reviewed and approved by Animal Care and Use Committee of Xi’an Jiaotong University.

## Author contributions

HF, HY, and D-JH conceived and designed the study. HF and D-JH screened participants for study entry. HF, ZC, H-BT and Z-YC processed the data. HF, ZC, L-QS, S-CL, Y-YZ, and X-YG collected samples and data. HF, ZC, H-BT and Z-YC performed experiments and analysis. HF, ZC, HY, and D-JH prepared the manuscript. All authors contributed to the article and approved the submitted version.

## Funding

This work was supported by grants from the National Natural Science Foundation of China (NSFC, Grant code: 81830077) to D-JH, NSFC (Grant code: 82171471) to HF, NSFC (Grant code: 82101551) to H-BT, NFSC (Grant code: 82071551) to HY, Shaanxi Natural Science Basic Research Program (2021JZ-39) and ShaanXi Nova Program(2022KJXX-54) to HF, Shaanxi Natural Science Basic Research Program (2021JQ-932) to H-BT, and China Postdoctoral Science Foundation (Grant code: 2018M643703, 2020T130526) to HF.

## Acknowledgments

We thank the support from Translational Medicine Center and Department of Spine Surgery of Hong Hui Hospital, and Department of Neurology, The Second Affiliated Hospital, Xi’an Jiaotong University.

## Conflict of interest

The authors declare that the research was conducted in the absence of any commercial or financial relationships that could be construed as a potential conflict of interest.

## Publisher’s note

All claims expressed in this article are solely those of the authors and do not necessarily represent those of their affiliated organizations, or those of the publisher, the editors and the reviewers. Any product that may be evaluated in this article, or claim that may be made by its manufacturer, is not guaranteed or endorsed by the publisher.
