# Supplementary material for: Necroptosis of nucleus pulposus cells involved in intervertebral disc degeneration through MyD88 signaling
Source: Front Endocrinol (Lausanne). 2022 Sep 21;13:994307. doi: 10.3389/fendo.2022.994307 (PMC9532572; doi:10.3389/fendo.2022.994307)
Supplement: Supplementary file 1 [file Table_1.docx]

Demographic data of LDH patients

| Patients’ ID | Gender | Age | Level | Degree |
| --- | --- | --- | --- | --- |
| 1 | M | 62 | L4/5 | 6 |
| 2 | F | 49 | L5-S1 | 6 |
| 3 | F | 49 | L4/5 | 5 |
| 4 | M | 45 | L4/5 | 6 |
| 5 | M | 45 | L4/5 | 6 |
| 6 | M | 44 | L4/5 | 5 |
| 7 | M | 57 | L4/5 | 6 |
| 8 | M | 43 | L5-S1 | 6 |
| 9 | F | 48 | L4/5 | 6 |
| 10 | M | 49 | L4/5 | 6 |
| 11 | M | 55 | L4/5 | 6 |
| 12 | F | 51 | L4/5 L5-S1 | 5 |
| 13 | M | 44 | L5-S1 | 6 |
| 14 | F | 50 | L4/5 | 5 |
| 15 | M | 38 | L5-S1 | 5 |
| 16 | F | 66 | L4/5 | 6 |
| 17 | M | 59 | L4/5 | 5 |
| 18 | F | 56 | L4/5 | 5 |
| 19 | F | 52 | L4/5 | 6 |
| 20 | M | 57 | L4/5 L5-S1 | 5 |
