# Supplementary material for: Necroptosis of nucleus pulposus cells involved in intervertebral disc degeneration through MyD88 signaling
Source: Front Endocrinol (Lausanne). 2022 Sep 21;13:994307. doi: 10.3389/fendo.2022.994307 (PMC9532572; doi:10.3389/fendo.2022.994307)
Supplement: Supplementary file 2 [file Table_2.docx]

Demographic data of traumatic lumbar fractures patients

| Patients’ID | Gender | age | Level | Degree |
| --- | --- | --- | --- | --- |
| 1 | F | 20 | L1/2 | 1 |
| 2 | M | 19 | L2/3 | 1 |
| 3 | F | 19 | L4/5 | 2 |
| 4 | M | 14 | L1/2 | 2 |
| 5 | F | 13 | L4/5 | 1 |
| 6 | M | 32 | L1/2 | 2 |
| 7 | F | 18 | L1/2 | 1 |
| 8 | M | 24 | L5-S1 | 2 |
| 9 | M | 30 | L5-S1 | 2 |
